# Partial staple line reinforcement with synthetic buttressing material in laparoscopic sleeve gastrectomy: a propensity score-matched analysis

**DOI:** 10.1007/s00423-023-02796-6

**Published:** 2023-01-20

**Authors:** Christoph Eckharter, Nickolaus Heeren, Francesco Mongelli, Martin Sykora, Julia Mühlhäusser, Nathalie Lottenbach, Andreas Scheiwiller, Jürg Metzger, Jörn-Markus Gass

**Affiliations:** 1 Department of General and Visceral Surgery, Lucerne Cantonal Hospital, Lucerne, Switzerland; 2 Department of Surgery, Lugano Regional Hospital, Lugano, Switzerland; 3 Department of Surgery, Nidwalden Hospital, Stans, Switzerland; 4 Department of Health Sciences and Medicine, University of Lucerne, Lucerne, Switzerland

**Keywords:** Obesity, Bariatric surgery, Laparoscopic sleeve gastrectomy, Staple line reinforcement, Outcomes

## Abstract

**Purpose:**

Staple line leakage (SLL) and staple line bleeding (SLB) are the most relevant postoperative complications of sleeve gastrectomy (SG). It is controversial whether and which method of staple line reinforcement (SLR) can best reduce these complications. The primary objective of this study was to investigate whether reinforcement of the most proximal part of the staple line with synthetic buttressing material, a strategy we termed partial SLR (p-SLR), reduces the 30-day incidence of SLL.

**Methods:**

A retrospective search of medical records of all bariatric patients from 2010 to 2019 was performed. Patients who underwent SG with either p-SLR or non-SLR were included. Intraoperative and postoperative outcomes were analyzed before and after propensity score matching (PSM).

**Results:**

Data from 431 patients were analyzed (364 in the p-SLR group and 67 in the non-SLR group). No difference in the 30-day incidence of SLL was observed between the two groups. The 30-day incidence of SLB (1.1% vs. 6.0% in the p-SLR and non-SLR groups, respectively) was significantly lower in the p-SLR group. These results were confirmed by PSM analysis.

**Conclusion:**

Partial staple line reinforcement with synthetic buttressing material does not reduce the 30-day incidence of SLL. Although our analysis showed a significant reduction in the 30-day incidence of SLB in the p-SLR group, this result should be interpreted with caution.

## Introduction

Sleeve gastrectomy (SG) is the second most commonly performed bariatric procedure in Switzerland. According to the Swiss Society for the Study of Morbid Obesity and Metabolic Diseases (SMOB), it accounted for about one-fifth of all bariatric surgeries between 2015 and 2020 [1]. Compared to Roux-en-Y gastric bypass, it seems to be a less technically demanding and time-consuming procedure. Although the overall complication rate is rather low, staple line leakage (SLL) and staple line bleeding (SLB) can be life-threatening and are the most feared complications after SG. A recent meta-analysis showed an incidence of SLL and SLB of 1.6% and 3.7%, respectively [2].

To avoid these complications, various strategies of staple line reinforcement (SLR) have been investigated. Examples include staple line oversewing, reinforcement with synthetic or biologic buttressing material, and the use of sealants (e.g., fibrin). The beneficial effects of SLR are controversial in the literature, and there is no consensus on which method is preferable [3–6].

Because the most proximal portion of the staple line close to the ankle of His is known to be the most common site for SLL [7], we hypothesized that a strategy of partial SLR (p-SLR) would reduce the rate of SLL. P-SLR includes reinforcement of the most proximal part of the staple line with GORE® SEAMGUARD® and represents an ideal compromise between the rather expensive reinforcement of the entire staple line and securing the weakest part at the ankle of His. GORE® SEAMGUARD® is a bioabsorbable synthetic buttressing material made from a synthetic copolymer — polyglycolic acid:trimethylene carbonate (PGA:TMC) — proven to reduce postoperative leaks and bleeding in SG and other minimally invasive surgeries [8–10]. To our knowledge, only two groups [11, 12] followed a similar strategy of p-SLR with GORE® SEAMGUARD® and failed to demonstrate a reduction in SLL and SLB.

## Material and methods

A retrospective search was performed in the medical records of all bariatric patients who underwent SG at a single bariatric surgery center (Obesity Center Central Switzerland at the Cantonal Hospital Lucerne) from January 2010 to December 2019.

Demographic and preoperative clinical data such as age, sex, body mass index (BMI), obesity-related comorbidities, and data on intraoperative and postoperative outcomes (operative time, intraoperative complications, length of hospital stay (LOS), complications within the first 30 days after surgery) were collected for all patients.

All patients for whom complete data were available and who underwent SG with p-SLR or without SLR (non-SLR) were included in the analysis.

The primary endpoint was the incidence of SLL within 30 days after surgery. SLL was defined as computed tomographic evidence of a fistula or abscess at the staple line or detection of a leak during revisional surgery when a complication was clinically suspected. Secondary endpoints were the incidence of SLB within 30 days after surgery, the incidence of intraoperative and postoperative complications other than SLL and SLB, operative time, and LOS. SLB was defined as active bleeding or the presence of hematoma as a stigma for recent bleeding from the staple line during revisional surgery or endoscopy.

### Surgical technique

In our bariatric surgery center, reinforcement of the most proximal part of the staple line with GORE® SEAMGUARD® was introduced in early 2012 as part of ongoing efforts to prevent complications like SLL. Previously, from 2010 to 2012, the staple line was not reinforced.

All surgeries were performed laparoscopically according to international standards [4, 6], and all procedures were performed by three experienced bariatric surgeons. A 36-French gastric tube was used for calibration. The first linear stapler was placed approximately 4–6 cm proximal to the pylorus, and staple cartridges were used in the following order from the antrum to the fundus of the stomach: 1× Echelon® 60 mm green cartridge (4.1 mm open, 2.0 mm closed), 1× Echelon® gold cartridge (3.8 mm open, 1.8 mm closed), 2–3× Echelon® blue cartridge (3.6 mm open, 1.5 mm closed), and 1× Echelon® gold cartridge. In the p-SLR group, the last staple cartridge (Echelon® gold cartridge) was reinforced with GORE® SEAMGUARD®. Intraoperatively leak test was performed with methylene blue, and visible SLB was managed with metallic clips.

### Statistical analysis

Descriptive statistics were presented as absolute number and percentage for categorical variables and median with interquartile ranges (IQR) for continuous variables. The comparison of categorical variables was performed with the chi-squared test or Fisher’s exact test, while continuous variables were compared with the Mann-Whitney *U* test. A propensity score-matched (PSM) analysis with 1:4 ratio was carried out according to gender, age, BMI, and comorbidities to minimize the effect of confounders [13]. A *p* value < 0.05 was considered statistically significant. Statistical analysis was performed on MedCalc® Statistical Software version 19.5.3 (MedCalc Software Ltd, Ostend, Belgium; https://www.medcalc.org; 2020).

## Results

**Fig. 1:**
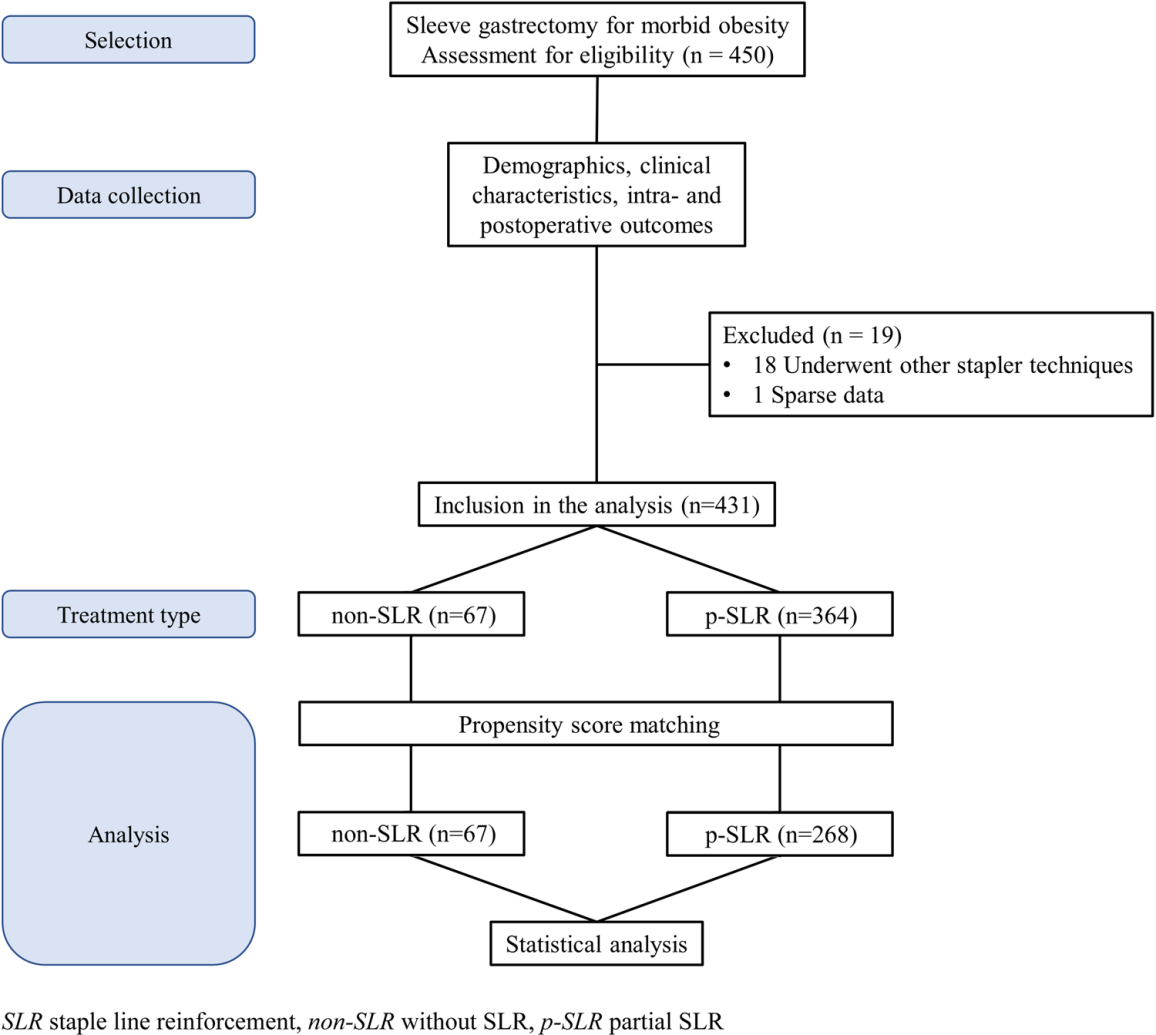
Flow chart for the inclusion of patients into the analysis

From January 2010 to December 2019, 431 of the 450 consecutive patients undergoing SG met the inclusion criteria and provided complete data (Fig. 1). The median age was 45.7 years (IQR 35.8–54.0), 172 patients (39.9%) were male, median BMI was 44.0 kg/m^2^ (IQR 38.5–50.2), and 361 (83.8%) had at least one comorbidity. No statistically significant difference was found in preoperative characteristics between the p-SLR and non-SLR groups (Table 1).

**Table 1:** Patient demographics before and after propensity score matching

	Unmatched analysis			PSM analysis		
	non-SLR (*n*=67)	p-SLR (*n*=364)	*p*	non-SLR (*n*=67)	p-SLR (*n*=268)	*p*
Age, years (IQR)	45.6 (35.5–52.2)	44.4 (33.0–54.9)	0.941	45.6 (35.5–52.2)	43.8 (31.9–54.9)	0.783
Male sex, *n* (%)	29 (43.3)	143 (39.3)	0.540	29 (43.3)	107 (39.9)	0.617
BMI, kg/m^2^ (IQR)	44.7 (39.9–50.5)	43.7 (39.3–50.4)	0.531	44.7 (39.9–50.5)	44.0 (39.5–51.1)	0.914
Diabetes, *n* (%)	22 (32.8)	108 (29.7)	0.604	22 (32.8)	76 (28.4)	0.472
Hypertension, *n* (%)	41 (61.2)	203 (55.8)	0.411	41 (61.2)	147 (54.9)	0.350
OSAS, *n* (%)	31 (46.3)	161 (44.2)	0.758	31 (46.3)	115 (42.9)	0.620
Hyperlipidemia, *n* (%)	21 (31.3)	102 (28.0)	0.580	21 (31.3)	72 (26.9)	0.465
GERD, *n* (%)	23 (34.3)	100 (27.5)	0.254	23 (34.3)	74 (27.6)	0.279
Asthma/COPD, *n* (%)	10 (14.9)	35 (9.6)	0.192	10 (14.9)	28 (10.4)	0.302
Previous abdominal surgery, *n* (%)	13 (19.4)	86 (23.6)	0.451	13 (19.4)	61 (22.8)	0.554

*PSM* propensity score matching, *SLR* staple line reinforcement, *non-SLR* without SLR, *p-SLR* partial SLR, *BMI* body mass index, *GERD* gastroesophageal reflux disease, *COPD* chronic obstructive pulmonary disease. Values are presented as median with interquartile range (IQR) or absolute number with percentage in parentheses

All cases were performed laparoscopically, and no conversion to laparotomy was recorded. *p* SLR was used in 364 patients (84.5%). Regarding intraoperative outcomes, a statistically significant difference between the groups was found only for operative time (72 min (IQR 55–94) vs. 95 min (IQR 72–124) in the p-SLR and non-SLR groups, respectively, *p*<0.001), which was also confirmed in the PSM analysis (72 min (IQR 55–94) vs. 95 min (IQR 72–124), *p*<0.001). Five intraoperative complications occurred, 3 in the p-SLR group (one uncontrollable bleeding due to splenic injury requiring splenectomy, one calibration tube entrapment in the staple line that required revision of the staple line and one minor bleeding due to liver injury requiring hemostasis with bipolar sealer) and 2 in the non-SLR group (one minor bleeding from a trocar access requiring laparoscopic suturing of the fascia and one minor bleeding due to liver injury requiring hemostasis with bipolar sealer).

Postoperatively, 29 complications were recorded: twenty-two (6.0%) in the p-SLR group (8 SLL (2.2%), 4 SLB (1.1%), 2 surgical site infections (SSI), 1 dialysis shunt occlusion, 1 iatrogenic small bowel lesion, 1 patient who died of unknown cause, 3 patients with temporal dysphagia due to a swollen staple line, 1 upper urinary tract infection, and 1 cardiac patient who received a blood transfusion prophylactically because his hemoglobin level fell below 80 g/L) and 7 (10.4%) in the non-SLR group (2 SLL (3.0%), 4 SLB (6.0%) and 1 SSI). The only statistically significant difference between groups was found in the incidence of SLB in the unmatched analysis (4 (1.1%) vs. 4 (6.0%) in the p-SLR and non-SLR groups, respectively, *p*=0.023), which was also confirmed in the PSM analysis (3 (1.1%) vs. 4 (6.0%), *p*=0.032).

Finally, LOS was significantly shorter in the p-SLR group in both the unmatched (4 days (IQR 4–5) vs. 4 days (IQR 3–4), *p*<0.001) and PSM analysis (4 days (IQR 4–5) vs. 4 days (3–4), *p*<0.001). Details of intra- and postoperative outcomes are shown in Table 2.

**Table 2:** Intra- and postoperative outcomes before and after propensity score matching

	Unmatched analysis			PSM analysis		
	non-SLR (*n*=67)	p-SLR (*n*=364)	*p*	non-SLR (*n*=67)	p-SLR (***n***=268)	*p*
Operative time, minutes (IQR)	95 (72–124)	72 (55–94)	<0.001	95 (72–124)	72 (55–94)	<0.001
Intraoperative complications, *n* (%)	2 (3.0)	3 (0.8)	0.174	2 (3.0)	2 (0.7)	0.180
Postoperative complications, *n* (%)	7 (10.4)	22 (6.0)	0.187	7 (10.4)	17 (6.3)	0.245
≥ Dindo-Clavien grade 3, *n* (%)	6 (9.0)	19 (5.2)	0.229	6 (9.0)	16 (6.0)	0.378
SL-Leakage, *n* (%)	2 (3.0)	8 (2.2)	0.658	2 (3.0)	8 (3.0)	1.000
SL-Bleeding, *n* (%)	4 (6.0)	4 (1.1)	0.023	4 (6.0)	3 (1.1)	0.032
Mortality, *n* (%)	0	1 (0.3)	1.000	0	1 (0.4)	1.000
Length of hospital stay, days (IQR)	4 (4-5)	4 (3-4)	<0.001	4 (4-5)	4 (3-4)	<0.001

*PSM* propensity score matching, *SL* staple line, *SLR* staple line reinforcement, *non-SLR* without SLR, *p-SLR* partial SLR. Values are presented as median with interquartile range (IQR) or absolute number with percentage in parentheses

## Discussion

Our analysis revealed no difference in the 30-day incidence of SLL between the p-SLR and non-SLR group. However, the 30-day incidence of SLB was significantly lower in the p-SLR group. In addition, the operative time and LOS were significantly shorter in the p-SLR group. Otherwise, no differences were observed in intraoperative or postoperative outcomes.

### Staple line leakage

Our main finding that SLL rates are similar in the p-SLR and non-SLR groups is largely confirmed in the literature.

The studies by Dawani et al. and Albanopoulos et al. used a similar method of p-SLR [11, 12]. In the study by Dawani et al., three groups were compared: non-SLR, p-SLR with GORE® SEAMGUARD®, and reinforcement of the entire staple line with GORE® SEAMGUARD®. In contrast to our p-SLR group, Dawani et al. reinforced the staple line at both the fundus end and the pyloric end. They defined SLL as the presence of contrast material outside the gastric lumen during the postoperative gastrografin swallow test and could not demonstrate a difference in the occurrence of SLL. Albanopoulos et al. compared a p-SLR group with a group in which the entire staple line was reinforced with a continuous suture. In contrast to our study, where only the last staple cartridge was reinforced with GORE® SEAMGUARD®, Albanopoulos et al. reinforced on average the last 3 staple cartridges. SLL was defined in their study as the presence of methylene blue in a routinely left drain on a swallow test on the second postoperative day or leakage of contrast material on an upper gastrointestinal series or computed tomography scan. Three months postoperatively, no difference in the incidence of SLL was observed between the two groups.

Other working groups [14–18] have studied the effects of reinforcing the entire staple line with GORE® SEAMGUARD® and, with the exception of Durmush et al. [5], have been unable to demonstrate a reduction in the incidence of SLL. However, the comparison groups were heterogeneous. Thus, in some studies, reinforcement of the staple line was omitted, whereas in others, such as the study by Durmush et al., reinforcement of the staple line was performed with oversewing or fibrin glue.

We decided to use SLR only on the most proximal portion of the staple line, since SLL occur most frequently in this part [7]. Reinforcing the entire staple line with GORE® SEAMGUARD® would theoretically only increase the cost without further preventing the risk of SLL. However, the results of our analysis do not support this strategy of p-SLR.

If we look in detail at the SLL that occurred in our patients and divide them into proximal and distal SLL, we see that 3 proximal and 4 distal SLL occurred in the p-SLR group. One SLL in the p-SLR group and the 2 SLL in the non-SLR group cannot be classified because, despite computed tomographic evidence of an abscess, the site of the SLL could not be clearly identified during revisional surgery.

The most appropriate method to find out whether our strategy of p-SLR has an impact on the reduction of SLL would be to compare the difference in the occurrence of proximal SLL between the two groups. Unfortunately, because classification into proximal or distal SLL was not possible in the non-SLR group, we could not perform this subgroup analysis. We can only speculate whether such an analysis would have revealed a difference in the occurrence of SLL.

### Staple line bleeding

Our analysis showed a significantly lower incidence of SLB in the p-SLR group, which contradicts the existing literature.

Dawani et al. and Albanopoulos et al., whose surgical technique of p-SLR is most similar to ours, found no effect of p-SLR with GORE® SEAMGUARD® on the occurrence of SLB. However, Dawani et al. only studied intraoperative bleeding from the staple line and not the incidence of postoperative SLB. Of the other groups [5, 14, 16–18] that performed reinforcement of the entire staple line with GORE® SEAMGUARD®, only Gayrel et al. demonstrated a significantly lower rate of SLB compared to a non-SLR group. However, Gayrel et al. focused on a high-risk population in their study, and this finding may represent a type of selection bias.

Yong et al. [19] reported that direct bleeding from the staple line and bleeding from transected branches of the right gastroepiploic artery and short gastric arteries are the main causes of SLB. Based on the results of the study by Chakravartty et al. [20], who reported that most intraoperative bleeding occurs in the middle part of the staple line (second to fourth staple firing), it can be concluded that the most proximal part of the staple line is not the most common site for the manifestation of SLB, unlike SLL.

The non-SLR group represents our early experience with SG in our bariatric center, which is also reflected in the significantly shorter operative time in the p-SLR group, as described below. Therefore, we interpret the lower incidence of SLB in the p-SLR group as a result of the surgeons’ learning curve and increasing experience and better standardization in perioperative care in this patient cohort.

### Other intra- and postoperative findings

We observed a significantly shorter operative time and LOS in the p-SLR group. As intraoperative complications were similar in both groups, we interpret the shorter operative time in the p-SLR group to be related to the surgeons’ learning curve and better standardization of the operation. Otherwise, it is difficult to justify a relevant effect of SLR on operative time.

Similarly, the shorter LOS cannot be attributed to a lower rate of postoperative complications, as their frequency (i.e., complications with a grade ≥ 3 according to Dindo-Clavien [21]) was similar in both groups. Therefore, the shorter LOS in the p-SLR group should be attributed to the surgeons’ experience and safety acquired over the years rather than to SLR.

### Limitations

This is a retrospective analysis in which data were retrieved from medical records. For this reason, the sample size is limited. Because our data were not randomized and the non-SLR group had fewer subjects, we performed propensity score matching to minimize imbalance in baseline characteristics and to make the two groups as comparable as possible.

## Conclusion

In sleeve gastrectomy, partial reinforcement of the staple line with GORE® SEAMGUARD® did not reduce the incidence of leakage. Given the limitations of our study and the lack of studies adequately designed to answer our hypothesis, further investigation with a randomized controlled trial is needed. Although there was a trend toward less bleeding in the p-SLR group, the results may be biased by the surgeons’ learning curve and should be interpreted with caution.
